# Supplementary material for: Simulating Interclonal Interactions in Diffuse Large B-Cell Lymphoma
Source: Bioengineering (Basel). 2023 Nov 27;10(12):1360. doi: 10.3390/bioengineering10121360 (PMC10740451; doi:10.3390/bioengineering10121360)
Supplement: Supplementary file 1 [file bioengineering-10-01360-s001.zip › bioengineering-2705732-supplementary.pdf]

**Supplemental Materials - Simulating Interclonal Interactions in Diffuse Large B-Cell Lymphoma**

**Supplementary Figures**

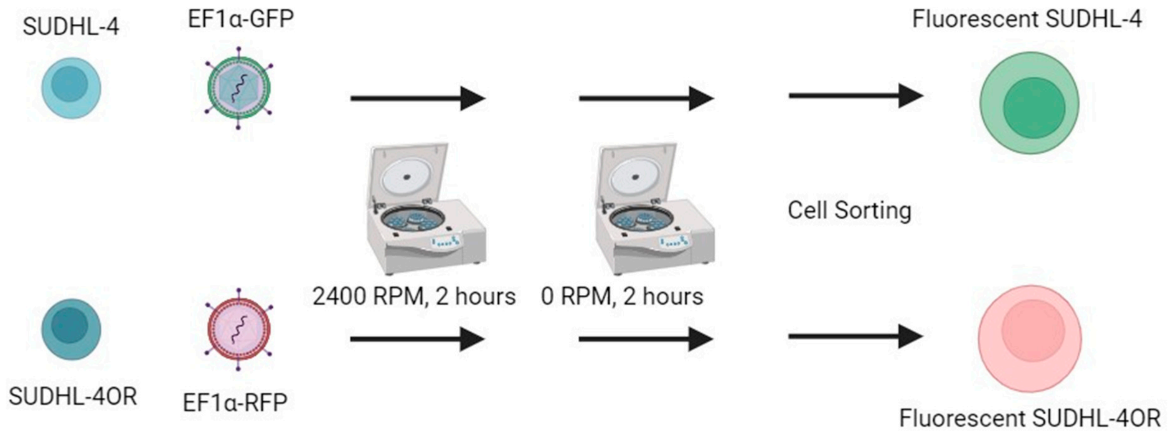


**Figure S1:** Process of spinoculation to create fluorescently-labeled DLBCL cells. Combined solutions of the SUDHL-4 clone and the respective viral particles are centrifuged for 2 hours, and kept within the centrifuge for 2 hours. The cells are then sorted to obtain cultures of pure fluorescent cells.

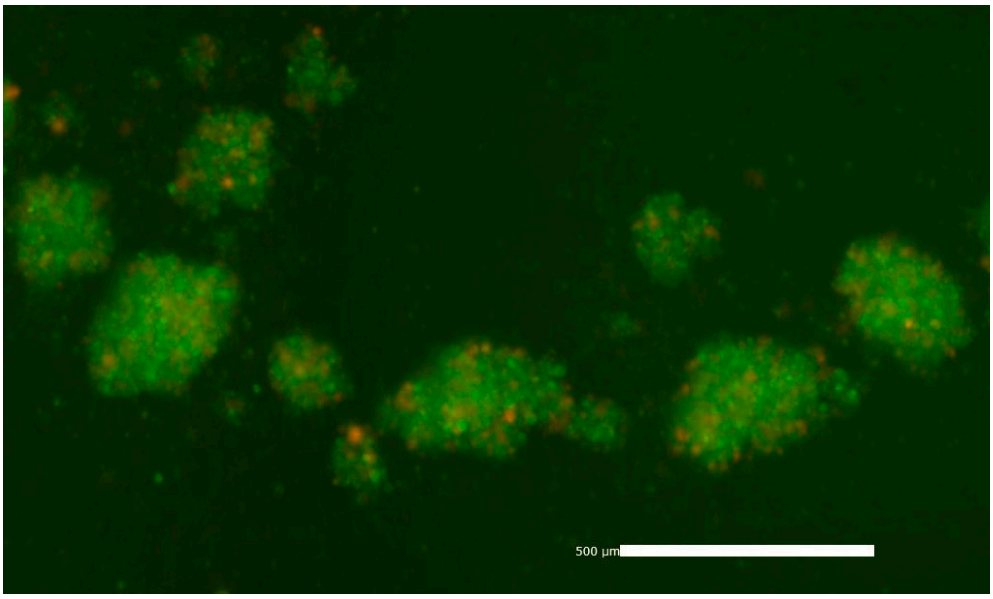


**Figure S2:** Aggregation of cells in mixed culture imaged with Celigo imaging cytometer - the aggregates contain SUDHL-4 (green) and SUDHL-4OR (red) cells.

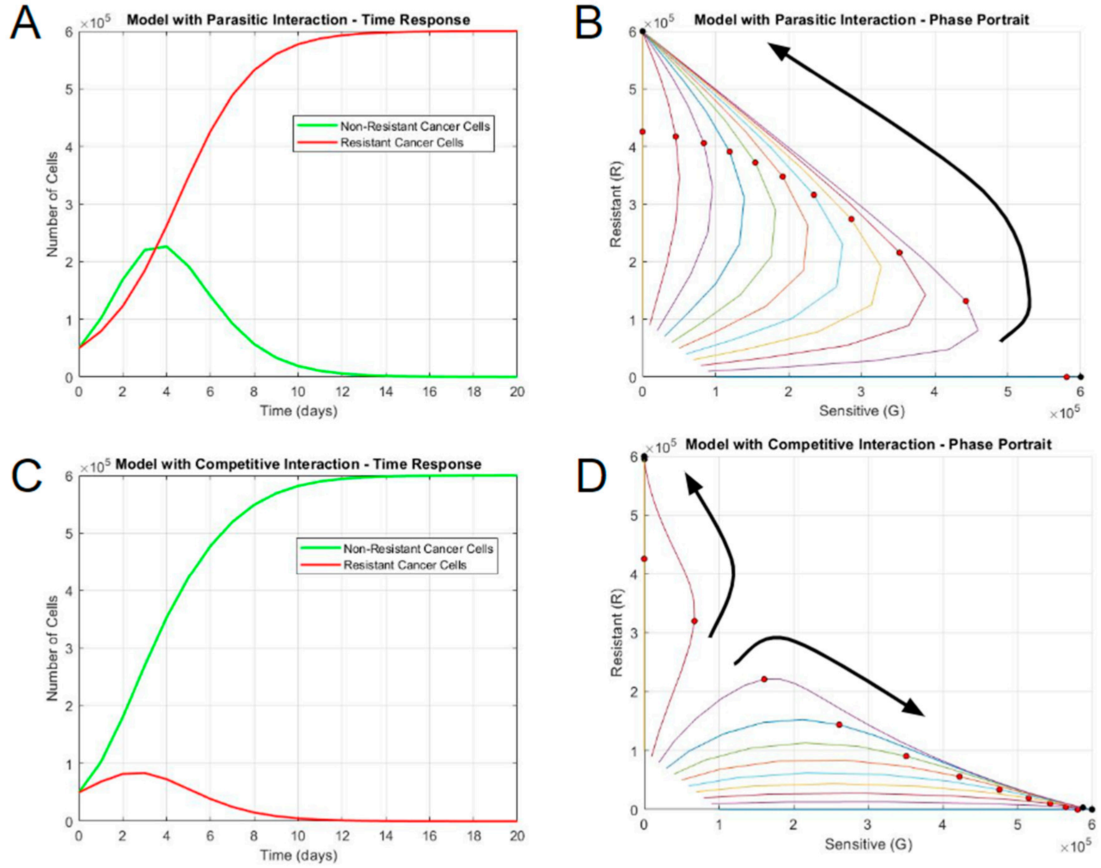


**Figure S3:** Long-term growth behaviors differentiate non-interacting and interacting cell populations. **A)** Time response and **B)** Phase portrait of logistic model with parasitic interaction. **C)** Time response and **D)** Phase portrait of logistic model with competitive interaction. In the phase portraits, the horizontal axis represents the number of sensitive DLBCL cells and the vertical axis represents the number of resistant DLBCL cells. Each curve in the phase portrait was created by altering the percentage of sensitive and resistant cells while keeping a constant initial population. The larger black arrows represent the general trajectories of population growth. The red circles represent the states on day 5, the black circle represent the steady state(s) at much longer time periods (day 100).

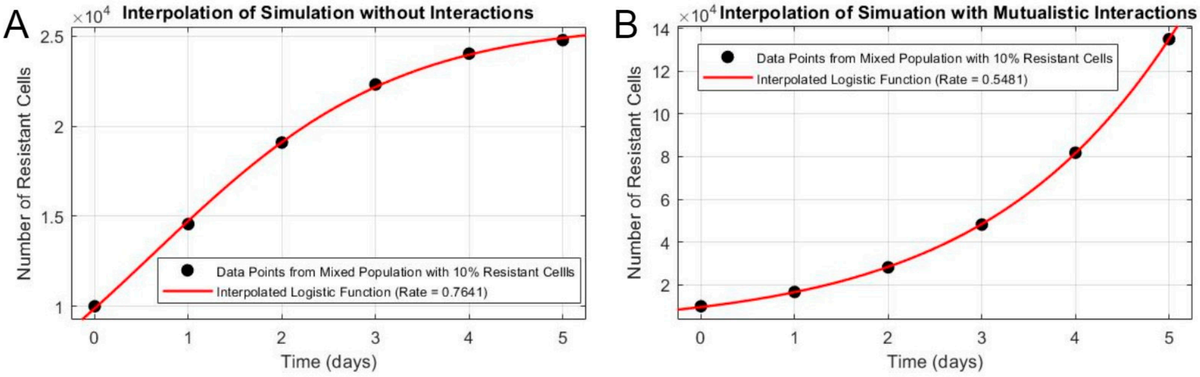


**Figure S4:** Example of rate constant interpolation from initial simulations. Sensitive and resistant cells from each simulation were separately fitted into a logistic equation to obtain rate constants ($k$). **A)** Non-interacting resistant cells in a 10% resistant cell culture demonstrate a rate constant of 0.7641 day$^{-1}$, whereas **B)** Interacting resistant cells in a 10% resistant cell culture demonstrate a rate constant of 0.5481 day$^{-1}$.

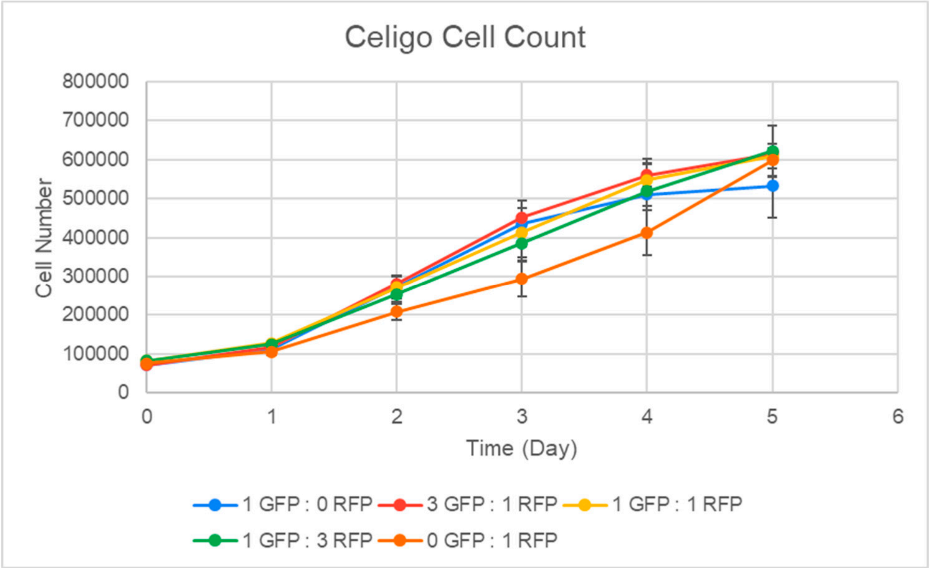


**Figure S5:** Total cell count of pure and mixed cultures from Celigo imaging cytometer. Cultures represent logistic growth.

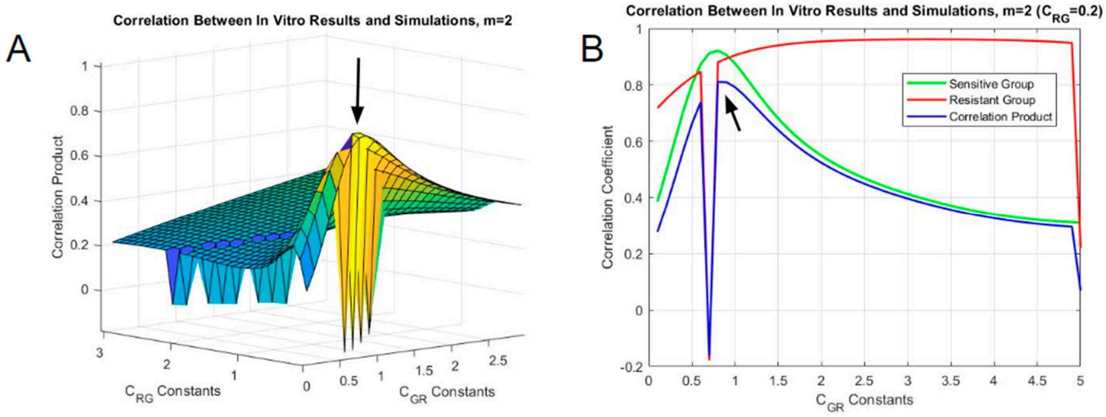


**Figure S6:** Optimization of the correlation product with **A)** 3-dimensional and **B)** 2-dimensional representations. The horizontal axes represent the range of tested interaction constants ($C_{GR}$ and/or $C_{RG}$). The vertical axis represents the correlation coefficients derived from correlating *in vitro* interpolated rate constants with rate constants from simulations using the indicated interaction coefficients. The correlation product is derived from multiplying correlation coefficients from the sensitive and resistant populations.

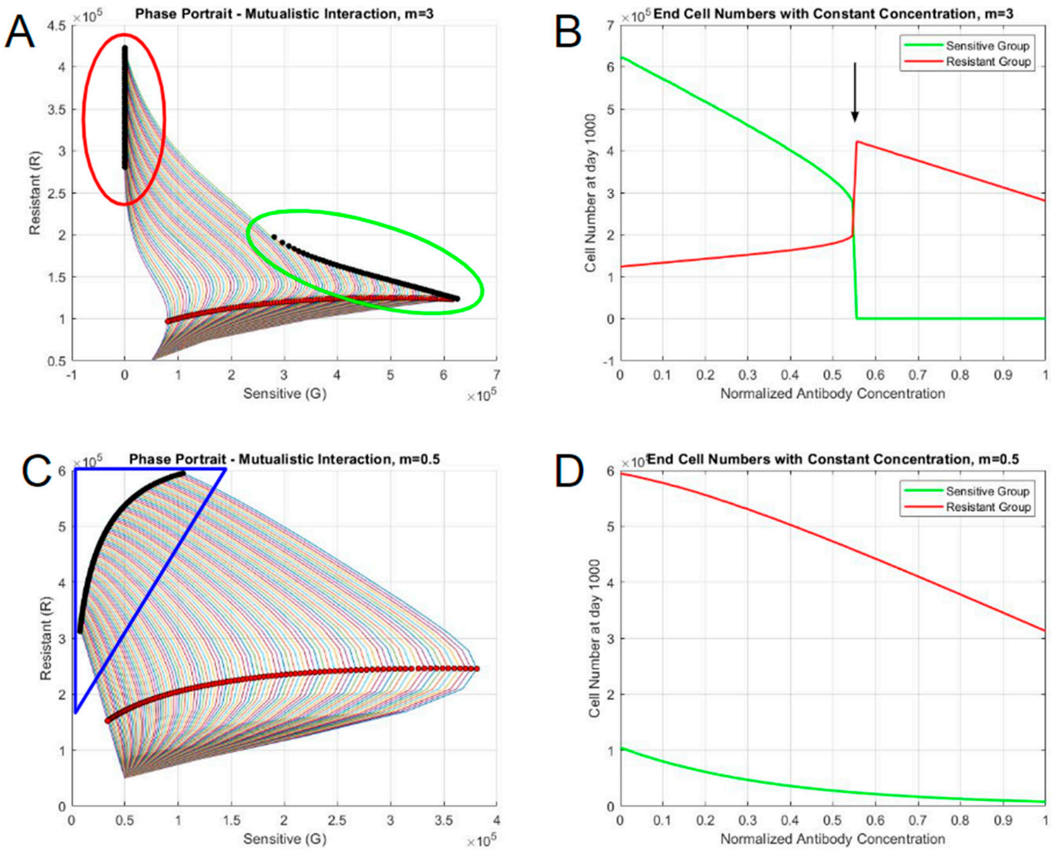


**Figure S7:** Steady state behaviors of systems with constant therapy. **A)** Phase portrait when m=3, **B)** End cell numbers when m=3, **C)** Phase portrait when m=0.5, **D)** End cell numbers when m=0.5. The red circles represent the states on day 5, the black circles represent the steady states. The outlines encircling sets of steady states represent distinct behaviors based on the concentration of therapy.

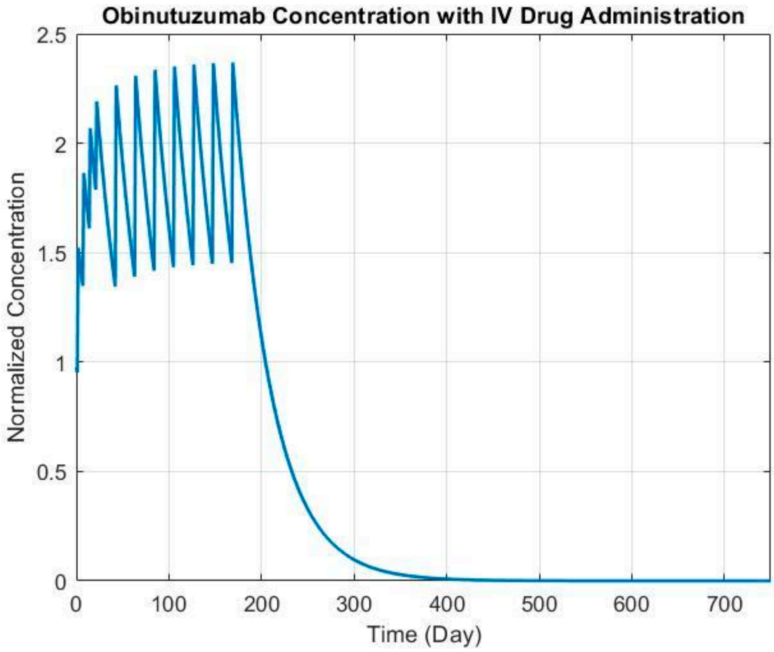


**Figure S8:** Obinutuzumab concentration over time based on therapy regimen. The vertical axis represents the concentration of therapy when the magnitude of the exponential function is set to one.

**Supplementary Tables**

**Table S1:** Interpolated constants from *in vitro* data

Green Fluorescent Protein (GFP) Constants

| | Initial Proportion of Cells | | | | |
|---|---|---|---|---|---|
| | 1 GFP : 0 RFP | 3 GFP : 1 RFP | 1 GFP : 1 RFP | 1 GFP : 3 RFP | 0 GFP : 1 RFP |
| k (Rate Constant, $day^{-1}$) | 1.069 | 1.133 | 1.191 | 1.065 | Not Applicable |
| CC (Carrying Capacity, number of cells) | 2.62E+05 | 2.72E+05 | 2.22E+05 | 1.81E+05 | Not Applicable |
| P(0) (Initial Population, Number of Cells) | 2.83E+04 | 2.38E+04 | 1.37E+04 | 8030 | Not Applicable |
| Adjusted $R^2$ | 0.9905 | 0.9934 | 0.9918 | 0.9985 | Not Applicable |

Red Fluorescent Protein (RFP) Constants

| | Initial Proportion of Cells | | | | |
|---|---|---|---|---|---|
| | 1 GFP : 0 RFP | 3 GFP : 1 RFP | 1 GFP : 1 RFP | 1 GFP : 3 RFP | 0 GFP : 1 RFP |
| k (Rate Constant, $day^{-1}$) | Not Applicable | 0.5128 | 0.6045 | 0.596 | 0.4695 |
| CC (Carrying Capacity, number of cells) | Not Applicable | 6.63E+04 | 7.57E+04 | 1.33E+05 | 3.10E+05 |
| P(0) (Initial Population, Number of Cells) | Not Applicable | 5401 | 8126 | 14000 | 22300 |
| Adjusted $R^2$ | Not Applicable | 0.9886 | 0.973 | 0.9488 | 0.9663 |

**Table S2:** Correlation products and interaction constants based on exponent value

| | | Interaction Coefficients ($day^{-1}cell^{-m}$) | |
|---|---|---|---|
| Exponent (m) | Correlation Product | $C_{GR}$ | $C_{RG}$ |
| 0.5 | 0.8078 | 0.08 | 0.2 |
| 1 | 0.7943 | 0.27 | 0.15 |
| 2 | 0.8105 | 0.8 | 0.2 |
| 3 | 0.8447 | 2.3 | 0.2 |

**Table S3:** Constants based on clinical data

Constants When the Interaction Exponent (m) is Equal to 2

| | *In Vitro* Constants | *In Vivo* Constants (Slow Growth) | *In Vivo Constants* (Fast Growth) |
|---|---|---|---|
| $k_G$ (Sensitive Cell Rate Constant, $day^{-1}$) | 1.068 | 0.0239 | 0.0495 |
| $K_R$ (Resistant Cell Rate Constant, $day^{-1}$) | 0.4695 | 0.0105 | 0.0218 |
| CC (Carrying Capacity, number of cells) | 6.00E+05 | 1.10E+12 | 1.10E+12 |
| $C_{GR}$ (Sensitive Cell Interaction Coefficient, $day^{-1}cell^{-2}$) | 0.8000 | 0.0179 | 0.0371 |
| $C_{RG}$ (Resistant Cell Interaction Coefficient, $day^{-1}cell^{-2}$) | 0.2000 | 0.0045 | 0.0093 |

Constants When the Interaction Exponent (m) is Equal to 1

| | *In Vitro* Constants | *In Vivo* Constants (Slow Growth) | *In Vivo Constants* (Fast Growth) |
|---|---|---|---|
| $k_G$ (Sensitive Cell Rate Constant, $day^{-1}$) | 1.068 | 0.0239 | 0.0495 |
| $K_R$ (Resistant Cell Rate Constant, $day^{-1}$) | 0.4695 | 0.0105 | 0.0218 |
| CC (Carrying Capacity, number of cells) | 6.00E+05 | 1.10E+12 | 1.10E+12 |
| $C_{GR}$ (Sensitive Cell Interaction Coefficient, $day^{-1}cell^{-1}$) | 0.2700 | 0.0060 | 0.0125 |
| $C_{RG}$ (Resistant Cell Interaction Coefficient, $day^{-1}cell^{-1}$) | 0.1500 | 0.0034 | 0.0070 |
